# Recharging Schedule for Mitigating Data Loss in Wireless Rechargeable Sensor Network

**DOI:** 10.3390/s18072223

**Published:** 2018-07-10

**Authors:** Haolin Liu, Qingyong Deng, Shujuan Tian, Xin Peng, Tingrui Pei

**Affiliations:** 1 College of Information Engineering, Xiangtan University, Xiangtan 411105, China; sjtianwork@xtu.edu.cn (S.T.); peitingrui@xtu.edu.cn (T.P.); 2 Key Laboratory of Intelligent Computing and Information Processing of Education Ministry, Xiangtan University, Xiangtan 411105, China; 3 Postdoctoral Research Station for Mechanics, Xiangtan University, Xiangtan 411105, China; 4 School of Information Science and Technology, Hunan Institute of Science and Technology, Yueyang 414000, China; pengxin@hnist.edu.cn

**Keywords:** wireless rechargeable sensor network, mobile charger, recharge schedule, criticality index, heuristic algorithm

## Abstract

Wireless Power Transfer (WPT) technology is considered as a promising approach to make Wireless Rechargeable Sensor Network (WRSN) work perpetually. In WRSN, a vehicle exists, termed a mobile charger, which can move close to sensor nodes and charge them wirelessly. Due to the mobile charger’s limited traveling distance and speed, not every node that needs to be charged may be serviced in time. Thus, in such scenario, how to make a route plan for the mobile charger to determine which nodes should be charged first is a critical issue related to the network’s Quality of Service (QoS). In this paper, we propose a mobile charger’s scheduling algorithm to mitigate the data loss of network by considering the node’s criticality in connectivity and energy. First, we introduce a novel metric named criticality index to measure node’s connectivity contribution, which is computed as a summation of node’s neighbor dissimilarity. Furthermore, to reflect the node’s charging demand, an indicator called energy criticality is adopted to weight the criticality index, which is a normalized ratio of the node’s consumed energy to its total energy. Then, we formulate an optimization problem with the objective of maximizing total weighted criticality indexes of nodes to construct a charging tour, subject to the mobile charger’s traveling distance constraint. Due to the NP-hardness of the problem, a heuristic algorithm is proposed to solve it. The heuristic algorithm includes three steps, which is spanning tree growing, tour construction and tour improvement. Finally, we compare the proposed algorithm to the state-of-art scheduling algorithms. The obtained results demonstrate that the proposed algorithm is a promising one.

## 1. Introduction

Wireless Sensor Network (WSN) is widely used in many applications, such as environmental monitoring, target tracking, security surveillance, etc. [1]. As nodes in a network are powered by energy-limited batteries, the network lifetime is always limited by nodes’ energy [2]. Owing to recent advances in Wireless Power Transfer (WPT) technology [3], a novel application named Wireless Rechargeable Sensor Network (WRSN) provides a promising approach to make sensor nodes work perpetually and has drawn growing attention from the research community [4,5,6,7,8].

In WRSN, when the battery of a node is depleted, a wireless charging vehicle called a mobile charger, which is equipped with an energy transceiver and high capacity battery, can move close to the node and charge it for a period of time. The maximal travel distance of the mobile charger is bounded due to its limited fuel capacity. After charging a number of nodes, the mobile charger has to return to the base station for refueling. Thus, due to the mobile charger’s refueling time and limited traveling speed, not every node which needs to be charged is serviced in time. In such a scenario, it is necessary to make a route plan for the mobile charger to determine which nodes should be charged first within the limited travel distance.

**Figure 1:**
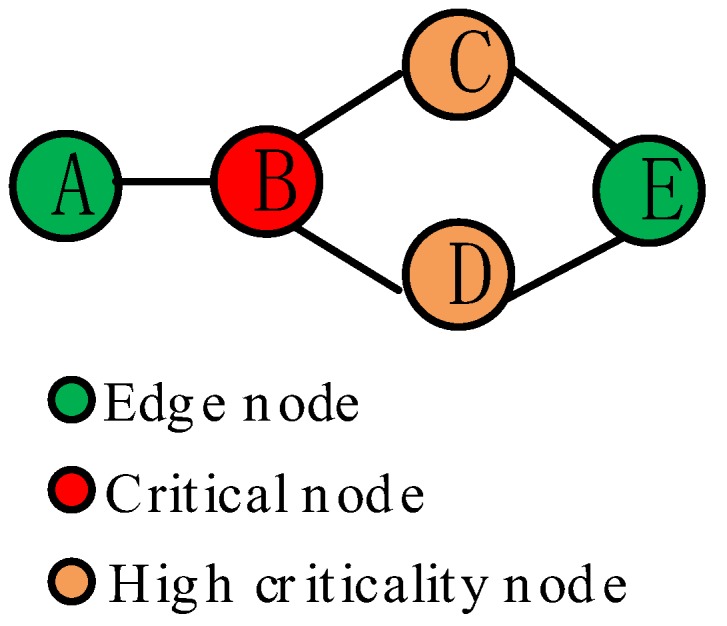
Example of high criticality nodes.

In WSN, a node usually behaves as both a data source and a data router [9,10,11]. The sensory data traffic follows a many-to-one pattern, where relay nodes tend to be congested as they are not only collecting and processing but also relaying data from nodes farther away. A node can be called critical node if it plays an irreplaceable role as a relay node in some routing paths. When a critical node depletes its energy, some other nodes that send their sensory data to the base station via this node can’t find an alternative routing path and become disconnected from the base station, which will make the network suffer data loss. In practical WSN, although the critical nodes are rare due to the dense deployment of nodes, there still exist some nodes that have a potential to be critical nodes, which are referred to as high criticality nodes in this paper. For example, as shown in Figure 1, there are the two paths A-B-C-E and A-B-D-E from node A to node E. Obviously, node B is a critical node that is irreplaceable in two paths. Node C and Node D are the high criticality nodes which will become critical node when the other is inactive. Thus, the high criticality node can also play a ‘bridging’ role in keeping the network connected, and the higher the criticality of a node is, the greater possibility to become critical node it owns. In order to decrease the risk of data loss, for the mobile charger, it is vital to charge as many high criticality nodes include critical nodes as possible before their batteries are depleted. To realize this, there is a question needed to be answered: How to quantify the criticality of nodes that the mobile charger can be scheduled to charge them effectively?

Betweenness centrality metric [12] is one of most commonly used direct measurement to characterize the criticality of nodes, by estimating the node’s contribution to the shortest routing paths construction. A node that participates in most of the shortest path is considered to be the highest criticality node. Since the betweenness centrality is computed by counting all of the paths through each node in the network, its computation is too costly to be adopted in the large network. Besides, the betweenness centrality of an edge node in the network is zero, which can’t determine the source node’s contribution. In the existing studies [13,14], to schedule the mobile charger, the node’s energy consumption rate is used as an indirect measurement of the node’s criticality. However, it is hard to accurately estimate node’s energy consumption rate in the practical WSN. Unlike previous metrics, a simple metric named criticality index is introduced in our work to quantify the criticality of nodes. The criticality index is the summation of node’s neighbor dissimilarity ratios. According to this index, a node is deemed as the high criticality node if it not only has many neighbors, but also is one of the few bridge nodes among its neighbors.

In this paper, a mobile charger is employed to charge the sensor nodes in WRSN. Due to the limited travel distance of the mobile charger, it can only serve a part of nodes during a single charging tour. The uncharged node will enter into a sleep mode when its battery is depleted and then wakes up after it gets charged. If the high criticality nodes become inactive, the network may suffer data loss. Therefore, in order to mitigate data loss in the network, we formulate an optimization problem with the objective of maximizing the sum of criticality indexes of nodes to be charged, subject to the mobile charger’s traveling distance constraint. Then, we propose a heuristic algorithm to solve this problem.

The main contributions of this paper are summarized as follows:

- To the best of our knowledge, this is the first work to consider the node’s disjointed state in the mobile charger scheduling problem. Compared to the state-of-arts, we distinguish the inactive nodes into two groups, the sleep nodes whose energy is exhausted and the disjointed nodes which are active but can’t send data to the base station via any available routing path. Most existing works only consider how to decrease the sleep node’s nonfunctional time with the help of a mobile charger, but ignore the disjointed node’s data loss. In our work, to maintain network connectivity as long as possible and mitigate the data loss, we consider reducing the inactive time of both sleep nodes and disjointed nodes via the mobile charger with limited charging ability.
- We introduce a metric called the criticality index to quantify the node’s criticality, which indicates a node’s contribution to network connectivity. It measures the dissimilarity between the node’s neighboring set and its neighbors’ neighboring sets. A node has a higher index if it plays a more important role as a bridge between two or more node sets. In addition, energy criticality is also considered, which indicates a node’s consumed energy ratio and can be used to measure the node’s desire for charging. We use this ratio to weight the criticality index to reduce the possibility of charging the full energy nodes. Otherwise, some higher criticality nodes will always be selected to be charged even if they have a great amount of residual energy.
- We formulate the charging scheduling problem as a novel optimization problem, with an objective of maximizing the total criticality indexes of nodes selected in the charging tour and subject to the mobile charger’s traveling distance constraint. To solve this NP-hard problem, we propose a heuristic algorithm. The heuristic algorithm includes three steps, which are spanning tree growing, tour construction, and tour improvement.

The rest of the paper is organized as follows. Section 2 gives brief overviews of the literature. In Section 3, we introduce the system model and define the problems. Section 4 proposes the heuristic algorithm for the charging scheduling problems. In Section 5, we conduct some simulations to compare the proposed algorithm with a state-of-art scheduling algorithm Nearest-Job Next with Preemption and a traditional Travel-Salesman-Problem based algorithm. Finally, we conclude this paper in Section 6.

## 2. Related Works

### 2.1. Wireless Power Transfer Technology

In this section, we mainly introduce three major technologies of WPT [15,16]: inductive coupling, electromagnetic radiation and magnetic resonant coupling.

Inductive coupling is driven by magnetic field induction [15]. Due to its simplicity and high-efficiency, it has been used in many applications and devices (e.g., medical implants, RFID tags, electric toothbrush). But it can’t be used to charge a sensor node in WRSN because it needs short charging distance and accurate alignment in charging direction.

Electromagnetic radiation [17], which is a radiative technology that transfers power on a radio frequency (RF), has the advantages of long distance power transfer. It can support the unidirectional and omnidirectional radiation. However, in unidirectional radiation, it requires Line-Of-Sight (LOS) and complex tracking mechanisms, leading to large scale of devices. And in omnidirectional radiation, its power transfer efficiency is very low.

Magnetic resonant coupling [3] works by having magnetic resonant coils operating at the same resonant frequency and generating a magnetic resonant induction for efficiently power transferring from a source coil to a receiver coil. Due to its high-efficiency and medium transfer distance, it becomes the most used WPT technology in WRSN [5,6,7,13,15,16]. For this reason, in the rest of this paper, we mainly focus on the magnetic resonant charging.

### 2.2. Mobile Charger Schedule

In recent years, a lot of efforts have been made on exploring the mobility-assisted energy replenishment in WRSN, in which the charging scheduling schemes for the mobile charger is a prominent issue. In general, charging scheduling schemes for WRSN can fall into two categories: Periodical scheme and on-demand scheme.

Periodical scheduling scheme converts the energy charging problem into a TSP based on nodes’ distribution model and energy consumption model, where the mobile charger knows all nodes’ statuses in advance and carries out the charging tour periodically. For example, Xie et al. [15] focused on the scenario where the mobile charger has to accomplish the charging tasks by visiting all the nodes in the network with the objective of maximizing the ratio of the mobile charger’s vacation time over the cycle time. The mobile charger’s shortest traveling path is decided in advance before its departing from the service station due to the energy consumption rates of nodes are unchanged. They [16] extended their work to a multiple-node charging case, in which the nodes around the mobile charger’s wireless power transmission range can be charged at the same time, thus greatly improving the charging efficiency. Fu et al. [18] proposed an optimal movement strategy for the mobile RFID reader to charge all nodes in the network, such that the charging delay is minimized. They used the concepts of the smallest enclosing space and a space discretization method to find the optimal stop locations for the mobile reader. They also a proposed energy synchronized mobile charging (ESync) protocol [19] with the assumption that nodes’ energy consumptions are already known by the mobile charger. They synchronized the charging request sequence of nodes with their sequence on charging tour to minimize the nodes’ charging delay and constructed a set of nested TSP tours involving the nodes with low residual energy to reduce the mobile charger’s moving distances. Guo et al. [20] proposed a framework of joint wireless energy replenishment and anchor-point based mobile data gathering in WRSN. They formulated the problem into a network utility maximization problem and presented a distributed algorithm to solve it. Shu et al. [21] formulated a mobile charger’s velocity control problem under the constraints of patrolling cycle and acceleration limit to maximize the network lifetime, and developed a near-optimal heuristic solution with a provable upper bound. Liang et al. [22] formulated a multiple mobile charger scheduling problem to charge life-critical sensors in the network with an objective to minimize the number of mobile chargers and then they proposed an approximation algorithm with a provable performance guarantee. The periodical scheduling schemes, which address the charging problem as an optimization path planning problem with the assumption that the node’s energy consumption rate is constant and known in advance by the mobile charger, are not suitable for the dynamic network, in which the node’s energy consumption rate is difficult to accurately estimate. 

In contrast, the on-demand scheduling scheme is carried out in an on-demand manner, where the mobile charger doesn’t know all nodes’ charging requests in advance. In practice, due to the dynamic variations of the node’s energy consumption, the mobile charger’s traveling path can’t be globally planned. In the on-demand scheme, node’s charging request is dynamically sent to the mobile charger only when the node’s residual energy falls below a predefined threshold. Then, the mobile charger will select one request to service and put others into a waiting queue. For example, He et al. [23] proposed an on-demand path planning method based on the discipline of Near Job Next with Preemption (NJNP), in which the mobile charger always greedily selects the nearest requesting node to charge. In Reference [24], Lin et al. proposed Double Warning thresholds with a Double Preemption (DWDP) charging scheme, in which double warning thresholds are used for activating the nodes’ charging requests. In Reference [25], a temporal and distantial priority scheduling method has been proposed for the on-demand charging architecture, in which distance between nodes and the mobile charger and arrival time of requests are considered to achieve better performance. The existing on-demand charging approaches adapt better to the variations in nodes’ energy consumptions compared to the periodical scheduling schemes, however they only address the problem for a single charging cycle without taking into account the fact that the mobile charger possesses limited traveling energy and has to return to the base station for energy replenishment periodically. Besides, the charging requests need to be sent to the mobile charger in time, which would occupy the channel resources.

## 3. Network Model and Charging Scheme

In this paper, we propose a scheduling algorithm based on the periodical scheme to meet the mobile charger’s energy replenish requirement. Unlike the aforementioned periodical approaches, the proposed scheduling algorithm does not depend on the accurate energy consumption rate of nodes, but takes into account the node’s criticality. In this section, we introduce the network model and criticality definition. At last, a novel criticality maximization problem is formulated.

### 3.1. Network Model

**Figure 2:**
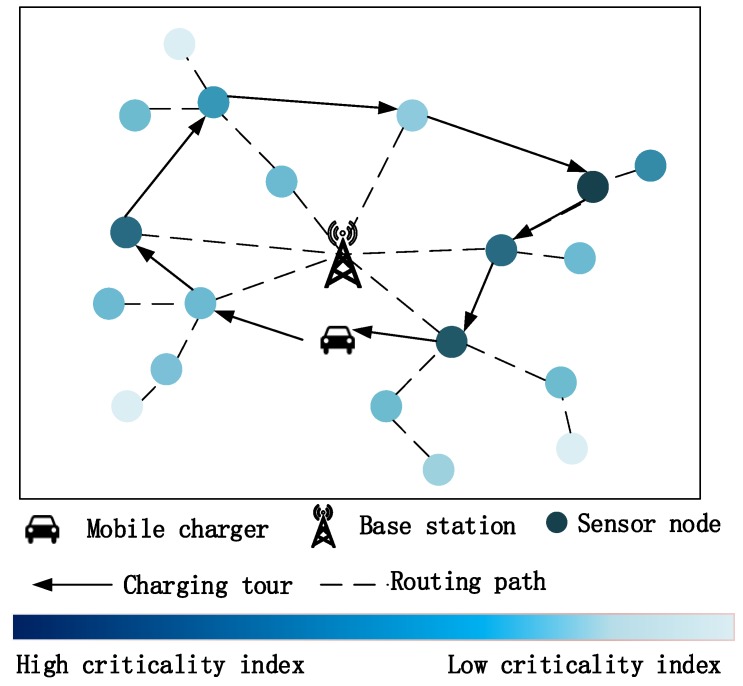
Illustration of the network architecture.

Figure 2 illustrates the network model in this paper. We consider a WRSN consisting of a set N={1,2,⋯,|N|} of sensor nodes randomly deployed in a rectangle area for environmental monitoring, where |·| denotes the cardinality of the set. A stationary base station is located at the center of rectangle area for data gathering and acts as a mobile charger’s depot. Each sensor node is powered by a rechargeable battery with a capacity of $E_{max}$ and consumes energy on sensing and wireless data transmission. We assume that each node uploads its sensory data to the base station via a routing path determined by a shortest multi-hop routing algorithm, e.g., Dijkstra’s routing algorithm. When the residual energy level of a node falls below $E_{min}$, the node will enter into a sleep mode and turn off most functions, including sensing and data transmission, until it gets the mobile charger’s energy replenishment.

To recharge the battery at each node, a mobile charger equipped with a powerful wireless energy transfer device is employed in the network. We assume that the mobile charger is driven by petrol or electricity, it can travel at a constant speed v and its traveling distance per tour is bounded by a given value L. The mobile charger is equipped with a high capacity battery that is sufficient to charge all sensor nodes on its charging tour. Due to the large scale of devices and the higher energy loss in the omnidirectional radiation pattern [13,26], in this paper, we adopt the point-to-point (direct) wireless power transfer pattern to maximize the energy wireless transfer efficiency. We assume the mobile charger charges only one node each time and each node will be fully-charged if the mobile charger visits, and the node’s received energy power $p_{r}$ is constant. During each tour, the mobile charger starts from the base station to charge nodes on its closed charging tour and returns before its traveling energy runs out. After finishing these point-to-point charging tasks, the mobile charger will stay at the base station to refuel or recharge itself for a period of time $T_{w}$. The major notations used in this paper are listed in Table 1.

### 3.2. Energy Consumption Model

In this paper, we assume the network is an event-driven network [6,13]. There are some events appear independently at random locations and random times in the network field. For simplicity, whenever an event appears in the sensing range $d_{s}$ of sensor nodes, these nodes consume $e_{s}$ energy to capture the event and transmit the sensory data to the base station via a dynamic routing path. Each node has the same maximum transmission range $d_{r}$, and consumes $e_{t}$ and $e_{r}$ energy to transmit and receive a data message, respectively.

### 3.3. Node’s Criticality Definition

In this work, the criticality is regarded as a quantification of node’s contribution in routing path construction, or connectivity maintenance. We introduce a metric called criticality index to measure node’s criticality.

For each node i, we define its neighbor sets as ${N_{b}}^{i}=\left\{j\in N|d(i,j)\leq d_{r},i\neq j\right\}$, where d(i,j) is the distance between node i and node j. The node i’s criticality index $r_{i}$ is given by:

(1) $$\psi_{ij}=\frac{\left|{N_{b}}^{j}|-|{N_{b}}^{j}\cap {N_{b}}^{i}\right|}{\left|{N_{b}}^{j}\right|}$$

(2) $$r_{i}=\sum_{j\in {N_{b}}^{i}}\psi_{ij}$$

where $\psi_{ij}$ is the dissimilarity ratio, which is a normalized indicator that takes a value between 0 and 1 to measure the difference between node i and node j’s neighbor sets. If node i and node j have a large number of common neighbors, the dissimilarity ratio $\psi_{ij}$ is small and, on the contrary, $\psi_{ij}$ is large if node i and node j have little common neighbors.

**Figure 3:**
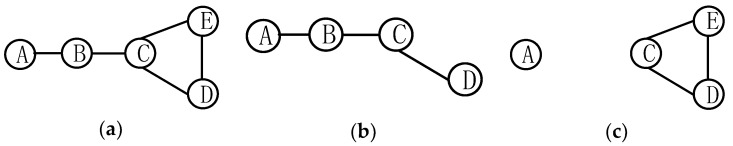
Example of network. (**a**) The original network; (**b**) The network of node E removed; (**c**) The network of node B removed.

The criticality index $r_{i}$ is calculated by summing the dissimilarity ratios of node i’s neighbors, which can be treated as an indicator of the node’s contribution in network connectivity. As such, the criticality index is determined by the node’s degree and the diversity of its neighbors. Firstly, if a node has higher node degree, it would have more neighbors and own greater possibility to become a relay node in numerous shortest routing paths than other nodes with a lower degree, which implies that the node owns higher criticality in connectivity maintenance. Besides, if a node is an isolated node and does not have any neighbors from the beginning of the network, its criticality index is 0. Secondly, the greater dissimilarity between the node’s and its neighbors’ neighbor sets, the higher criticality the node owns. We use the example network of Figure 3a to explain this part. In this network, nodes B and E have the same node degree, however, they own different criticality. Node B has two neighbor nodes, A and C. Node A’s neighbor is node B, while node C’s neighbors are node B, D, and E, thus node B doesn’t have the same neighbor nodes with node A or C. Like node B, node E also has two neighbor nodes, C and D. According to Equation (2), the criticality index of node E is smaller than that of node B due to the fact that C and D have the same neighbor E. The link between node C and D enhances the redundancy of connections, thus if node E is removed from the network, shown in Figure 3b, node C and D will be still connected. On the contrary, shown in Figure 3c, if node B is removed, the network will be split into two parts, and then node A will become a disjointed node. It demonstrates that node with a higher diversity of neighbors has a higher influence in network connectivity, and it should be treated as a higher criticality node.

However, only considering the node’s connectivity contribution is not enough to make a proper charging schedule for the mobile charger, where the energy criticality is also an important indicator that should be considered. If we ignore the nodes’ charging demand and only care about the connectivity contribution, the node with great contribution in connectivity will always be charged even if their energy is full capacity, on the other side, the node with less contribution like the edge nodes will never get the chance to be charged, causing a coverage hole in the network. To avoid that, the energy criticality, which represents a normalized ratio of the node’s consumed energy to the full energy capacity, is adopted to weight the criticality index and is defined as below:

(3) $$\rho_{i}=\frac{E_{max}-E_{i}}{E_{max}-E_{min}}$$

(4) $${\hat{r}}_{i}=\rho_{i}r_{i}$$

where $E_{i}$ denotes the residual energy level of node i when the mobile charger starts a new trip; $\rho_{i}$ denotes the energy criticality and ${\hat{r}}_{i}$ denotes the weighted criticality index. Obviously, according to the Equation (4), a node will get the higher charging priority if it has both a lower residual energy and a higher connectivity contribution.

### 3.4. Problem Formulation

The mobile charger’s scheduling problem can be defined as follow. We model the location of potential charging tasks in terms of a complete graph G=(V,E). For achieving high charging efficiency, the charger can only charge one node each time and stay close to the node when performing charging task. For the sake of simplicity, we assume that the locations of the charging task are coinciding with locations of nodes. Therefore, the vertex $v_{i}\in V(0\leq i\leq |N|)$ is the location of node i, and $v_{0}$ is the base station’s location where the tour starts and ends. Each vertex $v_{i}$ has a positive reward, which is equal to the weighted criticality index ${\hat{r}}_{i}$ of node i. We assume each node can be visited only once at most, therefore, the reward at a node can be collected only once. $E=\{(v_{i},v_{j})\}$ denotes the set of edges and presents potential paths between tasks. We define binary variables $x_{i}\in \{0,1\}(0\leq i\leq |N|)$ to be 1 if $v_{i}$ is visited in the tour and 0 otherwise. Define binary variables $y_{ij}\in \{0,1\}(0\leq i,j\leq |N|)$ to be 1 if the edge from $v_{i}$ to $v_{j}$ is traveled and 0 otherwise. Each edge $\left(v_{i},v_{j}\right)$ has an edge cost $c_{ij}$, which represents the Euclidean distance between $v_{i}$ and $v_{j}$, and $c_{ij}=c_{ji}$.

There is only one mobile charger with traveling distance constraint L in the network, which may not be able to visit and perform charging task on entire nodes. To maintain the network connectivity as long as possible, the mobile charger should preferentially visit the nodes with higher weighted criticality index and lower traveling cost during each charging tour. In other words, the sum of weighted criticality indexes of the nodes selected to be visited in a charging tour should be maximized, which can be treated as a maximization problem subject to the total traveling distance of the mobile charger is upper bounded by L. We formulate the maximization problem as an integer programming problem, which is shown as follow:

(5) $$\max\;\sum_{i=1}^{\left|N\right|}x_{i}{\hat{r}}_{i}$$

Subject to

(6) $$\sum_{v_{j}\in V\backslash \{v_{i}\}}y_{ij}=x_{i}\;v_{i}\in V$$

(7) $$\sum_{v_{i}\in V\backslash \{v_{j}\}}y_{ij}=x_{j}\;v_{j}\in V$$

(8) $$\sum_{i\neq j}y_{ij}\cdot c_{ij}\leq L\;v_{i},v_{j}\in V$$

(9) $$2\sum_{v_{k}\in S}x_{k}\leq |S|(\sum_{v_{i}\in S,v_{j}\notin S}y_{ij}+\sum_{v_{i}\notin S,v_{j}\in S}y_{ij})\;S\subset V\backslash \{v_{0}\};|S|\geq 2$$

(10) $$x_{0}=1$$

(11) $$x_{i}\in \{0,1\}\;v_{i}\in V$$

(12) $$y_{ij}\in \{0,1\}\;(v_{i},v_{j})\in E$$

In the above formulation, constraint (6) and (7) ensure that each vertex is visited at most once, where\denotes the set difference. Constraint (8) guarantees the edges traveled by the mobile charger would not exceed the distance limit. Constraint (9) is a sub-tour elimination constraint [27], where S is an arbitrary subset of V. Constraint (10) states that the base station must be selected. Constraint (11) and (12) impose the decision variables $x_{i}$ and $y_{ij}$ to be 0–1 valued, respectively.

Let L denote the budget and ${\hat{r}}_{i}$ denote the vertex reward, this formulation can be regarded as a kind of traveling salesman problem with profits, which is also called orienteering problem [28]. The goal of the orienteering problem is to design a closed path, or a tour, to visit a subset of vertices that maximizes the total reward collected, which has ingredients of both the traveling salesman problem (TSP) and the knapsack problem and is proved to be NP-hard [29].

## 4. A Heuristic Algorithm

Due to the NP-hardness of our problem, in this section, we design a heuristic algorithm for obtaining a fast solution.

The optimization procedure of the traveling salesman with profits involves vertex subset selection and shortest tour path construction. Hence, we first attempt to select as larger vertex subset as possible to efficiently utilize the budget, and then find an optimal TSP tour on the induced sub-graph of the selected vertex subset.

Our algorithm consists of three major steps, which is explained as follows.

### 4.1. Spanning Tree Growing

In order to select as many vertices in V as possible with the minimum traveling cost and maximum reward, we first grow a tree using greedy knapsack approach. Suppose the spanning tree T starts from vertex $v_{0}$, and the tour distance, also called tour cost of the tree is C(T). By the fact from graph theory: given any spanning tree T, the cost of an optimal tour on the vertices is no greater than twice the cost of edges in the tree, the tour cost C(T) of selected vertices can be quickly estimated.

Firstly, to grow the tree T, for each unselected vertex $v_{i}\in V\backslash T$, the minimum cost that adding it to be a leaf node in the tree is computed and denotes $c_{i}$:

(13) $$c_{i}=\min_{v_{k}\in T}c_{ik}$$

where $c_{ik}$ denotes the distance between $v_{i}$ and $v_{k}$. It is possible that $c_{i}$ can be improved while $v_{i}$ is inserted as $v_{k}$’s parent node instead of the leaf node. Thus, the addition-cost when $v_{i}$ is inserted as $v_{k}$’s parent node is further computed, and the smaller cost is used to be $v_{i}$’s final addition-cost $c_{i}^{\prime }$.

(14) $$c_{i}^{\prime }=\min\{c_{i},\;c_{i}+c_{p(k)i}-c_{p(k)k}\}$$

where p(k) denotes $v_{k}$’s parent node.

Secondly, define $v_{i}$’s reward-to-addition-cost ratio:

(15) $$R(i,T)=\frac{{\hat{r}}_{i}}{c_{i}^{\prime }}$$

Finally, find the vertex $v_{i}$ with the biggest R(i,T) in the unselected vertices V\T whose addition cost satisfies $C(T\cup \{v_{i}\})\leq L$, and add it to the tree.

This procedure is iterated until no more vertices outside T can satisfy $C(T\cup \{v_{i}\})\leq L$. The detailed algorithm is described in Algorithm 1.
**Algorithm 1.** Tree growing.**Input:**G=(V,E), traveling distance constraint L;**Output:** spanning tree T1:$T:=\{v_{0}\}$2:**while**C(T)≤L and V\T≠ϕ
**do**3: R:=ϕ4: **for** each $v_{i}\in V$ and $v_{i}\notin T$
**do**5:  $c_{i}:=\underset{v_{k}\in T}{\min}\;c_{ik}$6:  $c_{i}^{\prime }:=\min\{c_{i},c_{i}+c_{p(k)i}-c_{p(k)k}\}$7:  $R(i,T):={\hat{r}}_{i}/c_{i}^{\prime }$8:  R:=R∪{R(i,T)}9: **end for**10: find the biggest R(i,T) in R11: $v_{i}:=\arg\;\max_{R(i,T)\in R}\;R(i,T)$12: $C(T\cup \{v_{i}\}):=2(c_{p(i)i}+\sum_{\begin{array}{l} v_{k}\in T,\\ k\neq 0 \end{array}}c_{p(k)k})$13: **if**
$C(T\cup \{v_{i}\})\leq L$
**then**14:  $C(T):=C(T\cup \{v_{i}\})$15:  $T:=T\cup \{v_{i}\}$16: **else**17:  break18:** end if**19:**end while**20:**return**T


### 4.2. Tour Construction

The spanning tree growing from the first step selects a set of vertices T that should be the charging nodes after the mobile charger starts its charging tour. To find the shortest path to complete the charging tour, we apply the Lin-Kernighan-Helsgaun (LKH) algorithm [29] on the selected vertices to construct an approximate TSP tour. The constructed tour path, also can be seen as the charging sequence is denoted by P, with cost $C_{\mathrm{LKH}}(P)$. If $C_{\mathrm{LKH}}(P)>L$, the last vertex added into T will be removed and the new tree will apply the LKH algorithm again. This procedure is iterated until $C_{\mathrm{LKH}}(P)\leq L$. The detailed algorithm is described in Algorithm 2.
**Algorithm 2.** Tour construction.**Input:** Spanning tree T, traveling distance constraint L;**Output**: A charging tour P starts from and ends at $v_{0}$1:**repeat**2: compute shortest path in the T,P:=LKH(T)3: **If**
$C_{\mathrm{LKH}}(P)\leq L$
**then**4:  Declare P is accepted5: **else**6:  Delete the last added node from T7: **end if**8:**until**P is accepted9:**return**P


### 4.3. Tour Improvement

It is possible to insert some unselected vertices into the tour P constructed by the LKH algorithm in the last step, whose distance does not exceed L. For each vertex $v_{i}\in V\backslash P$, let’s define its insertion cost as $c_{i}^{\prime \prime }=\min_{v_{x},v_{y}\in P}c_{ix}+c_{iy}-c_{xy}$, where $v_{x},v_{y}$ are adjacent vertices in P. If $C_{\mathrm{LKH}}(P)+c_{i}^{\prime \prime }\leq L$, then vertex $v_{i}$ is feasible for adding to the tour. Find the feasible vertex in a set V\P with highest reward-to-insertion-cost, which is computed as ${\hat{r}}_{i}/c^{\prime \prime }$, and then insert it into the tour at the location with lowest insertion cost. Repeat this procedure until no more feasible vertices in V\P can be inserted. The detailed algorithm is described in Algorithm 3.
**Algorithm 3.** Tour improvement.**Input: **G=(V,E), initial charging tour P, traveling distance constraint L;**Output:** An improved charging tour P1:**repeat**2: R:=ϕ3: **for** each vertex $v_{i}\in V$ and $v_{i}\notin P$
**do**4:  $c_{i}^{\prime \prime }:=\underset{v_{x},v_{y}\in P}{\min}\;c_{ix}+c_{iy}-c_{xy}$5:  **if**
$c_{i}^{\prime \prime }+C_{LKH}(P)\leq L$
**then**6:   $R(i,P):={\hat{r}}_{i}/c_{i}^{\prime \prime }$7:   R:=R∪{R(i,P)}8:  **else**9:   continue10:  **end if**11: **end for**12: $v_{i}:=\arg\;\max_{R(i,P)\in R}\;R(i,P)$13: insert $v_{i}$ into P at the location with minimum insertion cost14:**until** no more feasible vertices in V\P15:**return**P


### 4.4. Time Complexity Analysis

The time complexity of the proposed heuristic algorithm can be analyzed as follows.

The spanning tree growing algorithm can be treated as element insertions from a full set to an empty set, which requires 1×|N|+2×(|N|−1)+⋯+|N|×1=|N|(|N|+1)(|N|+2)/6 iterations in the worst case. Thus, it has time complexity $O(n^{3})$ [29]. The computation complexity of tour construction is dependent on that of LKH algorithm, which is $O(n^{2.2})$. The worst case is that all the nodes in the growing tree don’t meet the constraint of the tour distance, which means that the tour construction will iterate for |N| times at most and take $O(n^{3.2})$ time. The tour improvement is like the tree growing, which requires $O(n^{3})$ time in worst case. Hence, the time complexity of the proposed heuristic algorithm can be approximated as $O(n^{3})$.

## 5. Simulation Evaluations

In this section, we evaluate the performance of the proposed charging schedule through experimental simulation. The simulation is built in the widely-used OMNET++ simulator [30]. We also study the impact of important parameters on the performance, including the number of nodes deployed, the charger’s traveling distance constraint, the charger’s traveling speed and the node’s received charging rate.

### 5.1. Simulation Environment

We consider an event-driven sensor network deployed in a 100 m × 100 m square. The network consists of 100 sensor nodes, which are uniformly randomly distributed in the network. A base station is located at the center of the area. The node’s communication range is 25 m to form a multi-hop network topology and Dijkstra’s shortest path dynamic routing algorithm is used. In this routing algorithm, each node out of the one-hop distance to the base station would find an active node which is in its transmission range and closer to the base station, then sends sensory data to the base station via it. We assume that each node’s maximum battery capacity is 1000 J by equipping a 138 mAH battery with a typical voltage of 2 V. The transmitting and receiving costs of nodes are set based on the MICA2’s datasheet: the node’s energy consumption rates on transmitting and receiving are 25 mA × 2 V = 50 mW and 8 mA × 2 V = 16 mW, respectively [19]. With the sensory data packet is in the size of 120 bytes and the bit rate of 9.6 Kbps [31], each node approximately consumes 5 mJ and 1.6 mJ in transmitting and receiving one sensory data packet, respectively. In the event-driven network, whenever events occur in the nodes’ sensing range, these nodes would consume 0.15 mJ energy to capture the event [19], then generate a sensory packet and transmit it to the base station via the routing path. The relay nodes on the routing path would apply the data aggregation technique to combine the correlated sensory data. We assume that the mobile charger travels at a constant speed of 1 m/s [19]. Considering the advance in the WPT technology based on the strongly magnetic resonances [3,26], we assume the mobile charger can replenish energy to the sensor nodes at a rate of 5 W. The mobile charger’s traveling distance constraint is 600 m [20]. To simplify the presentation, we set $E_{min}$ equals to 0. We set the simulation time to 100,000 s and record each node’s disjointed time and sleep time. We repeat the simulation 50 times with different network distribution and calculate the mean of total disjointed duration, inactive duration and data loss rate as the results. The total disjointed duration is defined as the total time of the nodes which become disjointed and can’t send any sensory data to the base station via a dynamic routing path. Although the node in sleep mode is also disjointed from the base station, we only consider the active node’s disjointed time. Thus, the total disjointed duration is positively correlated with the high criticality nodes’ sleep time, which can reflect the charging priority of the high criticality nodes in the scheduling algorithm. Considering the node’s sleep time, the node’s total inactive time is the union of disjointed time and sleep time, which can reflect the charging effect to all of nodes in the scheduling algorithm. The default parameters set in the simulation is shown in Table 2.

### 5.2. Property Analysis

To verify the assumption on the criticality index of the node, we conduct an experiment based on the default parameters mentioned above.

**Figure 4:**
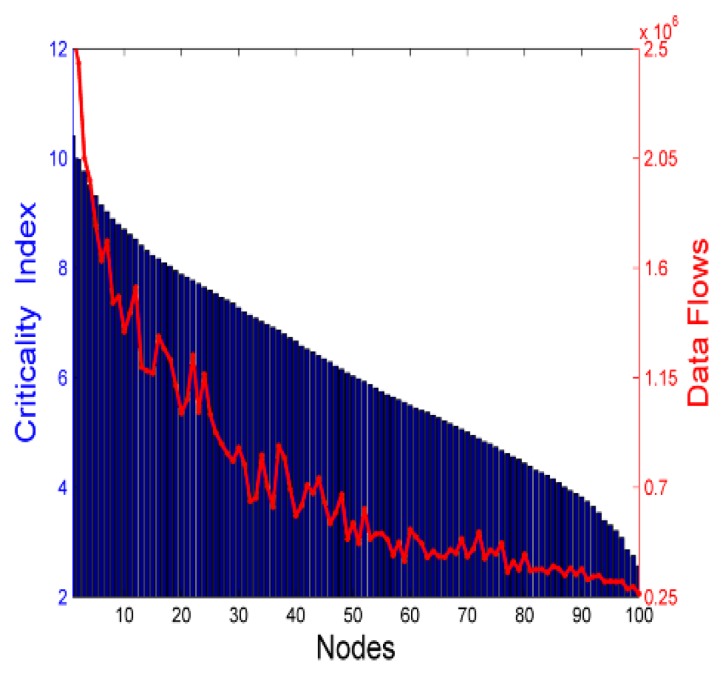
Trend chart of criticality index and data flows.

The verification result is shown in Figure 4, where the bar denotes the sorted node’s criticality index and the curve denotes the data flows generated and relayed by the related node. If a node has higher data flows, which implies that the node plays an important role in data collection or data relaying, it would also have higher criticality index in our assumption. The data flows can be more accurate to measure the connectivity contribution of a node, but this value is difficult to obtain during the network’s running time. In contrast, the criticality index can be calculated after the network deployment and kept unchanged. From Figure 4, we can observe the correlation between the node’s criticality index and its data flows. Except for some mismatch cases, the overall trend of data flows decreases as the criticality index decreases, which demonstrates that the index can be used to evaluate the node’s connectivity contribution. 

### 5.3. Performance Comparison

We mainly contrast our scheduling algorithm with the well-known NJNP and TSP algorithm. In NJNP, the mobile charger would service the nearest nodes, which have sent a charging request with the preemption on-demand mode, and move back to the base station when its traveling path distance is approaching the limitation. In TSP, the mobile charger would select the lowest-energy nodes in the network and use the LKH algorithm to compute the shortest path to charge them within the traveling distance constraint, which can be treated as a tour construction algorithm only consider energy criticality. To verify the performance of criticality index as the node’s criticality measurement and illustrate the necessity of combining the consumed energy ratio into the weighted criticality index, we contrast our heuristic algorithm with the different node’s rewards, which are the proposed weighted criticality index (WCI), the criticality index without consumed energy ratio (CI) and betweenness centrality (BC), respectively.

#### 5.3.1. Impact of Network Scale

**Figure 5:**
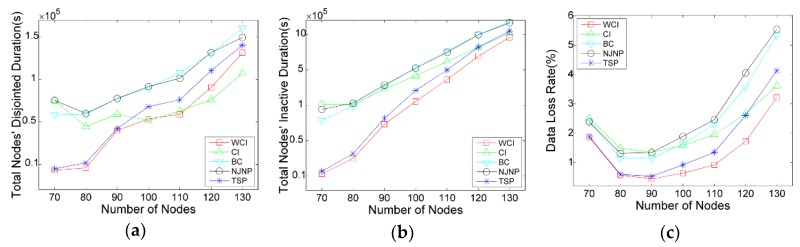
Impact of |N| on the charging scheduling algorithms: (**a**) Total nodes’ disjointed time; (**b**) Total nodes’ inactive time; (**c**) Network’s data loss rate.

We first evaluate the charging scheduling algorithm’s performance with respect to the number of deployed nodes, which vary from 70 to 130. Another simulation parameters are set as default mentioned above. The results are shown in Figure 5a–c, respectively.

Figure 5 illustrates performance curves of the algorithms. We can see that with the number of nodes deployed in the network increases, both disjointed and inactive time grow rapidly. For example, the total nodes’ disjointed duration and total nodes’ inactive duration of WCI are, respectively, about 8521 s and 10,776 s when 70 nodes are deployed, but these two durations are increased to, respectively, about 207,204 s and 913,592 s when 130 nodes are deployed. This is due to the increase of |N|, the burden of the relay nodes, especially the neighbor nodes of the base station, are significantly increasing, leading to the frequent exhaustion of those nodes and the increase of disjointed and inactive time of the network. There exists a decreasing tendency of the network’s data loss rate from |N|=70 to |N|=90. This is because when |N|=70, the nodes are deployed sparsely, which causes the poorer the network connectivity and the more the data have been lost. When |N|=90, an increasing tendency of the network’s data loss rate exists, where the data loss is mainly caused by the relay nodes’ frequent exhaustion. From the result, we can see that the total nodes’ disjointed time, inactive time and networks data loss rate by WCI outperform the NJNP and TSP in all the cases. For example, when |N|=100, the total disjointed time of network by WCI is 72%, the total inactive time is 70%, and the data loss rate is 69% of that delivered by the TSP. In contrast, when compared with NJNP, the three ratios are reduced to 42%, 34%, and 34%, respectively. Compared to WCI, CI has inferior performance in most cases. This is because that CI ignores the node’s residual energy, leading to the result that the nodes with a little contribution in connectivity are rarely visited even if their batteries are depleted, which increases the inactive nodes’ number and then causes additional data loss in the network. Compared to WCI and CI, BC considers the betweenness centrality of nodes, in which only a few nodes have higher betweenness centrality while the other nodes are 0 or lower value. Unfortunately, some potential critical nodes and edge nodes among these nodes exist with lower betweenness centrality. Thus, the mobile charger only charges a few nodes in the BC scheduling algorithm, leading to the poorer network connectivity and severer data loss than WCI and CI.

#### 5.3.2. Impact of Charger’s Traveling Distance Constraint

**Figure 6:**
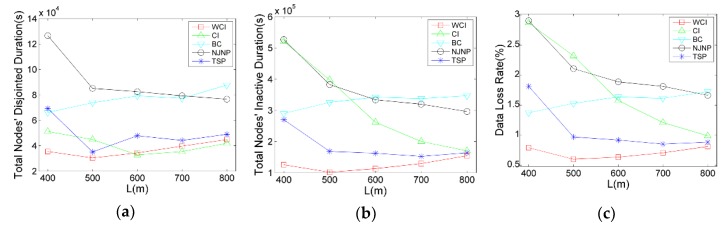
Impact of L on the charging scheduling algorithms: (**a**) Total nodes’ disjointed time; (**b**) Total nodes’ inactive time; (**c**) Network’s data loss rate.

Figure 6 shows the performance comparisons of NJNP, TSP, WCI, CI and BC with varying charger’s traveling distance constraint from 400 to 800 meters. The advantage of WCI can be clearly observed. For example, when L=600 m, WCI’s total disjointed time, inactive time and data loss rate is 72%, 70%, and 69% of that delivered by the TSP, respectively. When compared with NJNP, the three ratios are reduced to 42%, 34%, and 34%, respectively. The two durations and data loss rate delivered by the NJNP decrease as L increases, this is because the charger can visit more nodes and accomplish more charging tasks in charging tour if L increases. In contrast, the TSP and WCI don’t have any improvement on the results as the charger’s traveling distance increases. When L increases, the charger is able to charge more nodes but only once in a tour using the TSP and WCI, which makes some high-energy-consumption nodes charged in the early time of tour exhausted again and have to be waited for charging until the next tour. Meanwhile, the CI’s total inactive time and data loss rate are approaching to the results of TSP and WCI. This is because that the number of charged nodes in the tour increases and the difference of selected nodes between these algorithms is diminishing. Even so, the WCI still outperforms CI in most cases.

#### 5.3.3. Impact of Charger’s Traveling Speed

**Figure 7:**
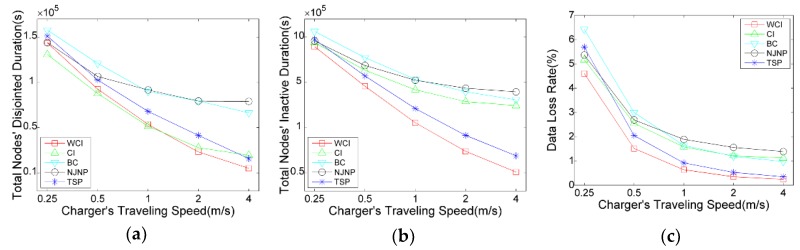
Impact of v on the charging scheduling algorithms: (**a**) Total nodes’ disjointed time; (**b**) Total nodes’ inactive time; (**c**) Network’s data loss rate.

Figure 7 shows the performance comparison of NJNP, TSP, WCI, CI and BC with varying charger’s travel speed. Figure 7 demonstrates that when v increases, the two durations and data loss rate significantly decrease. This is because the faster the charger travels, the shorter time it consumes in the travel path, and the more nodes can be charged in a tour. Compared with NJNP and TSP, we can see that WCI illustrates its advantage. For example, when v=1, WCI’s total disjointed duration, inactive duration, and data loss rate is 72%, 70%, and 69% of that delivered by the TSP, respectively. When compare with NJNP, the three ratios are reduced to 42%, 34%, and 34%, respectively. Note that CI outperforms WCI on total nodes’ disjointed duration when v is lower. This is because the connectivity critical nodes are charged first in the CI. Consequently, the total disjointed time of active nodes is shorter while the total inactive time of other nodes is longer, which implies the charging effect of CI is inferior to the WCI and TSP.

#### 5.3.4. Impact of Charging Power

**Figure 8:**
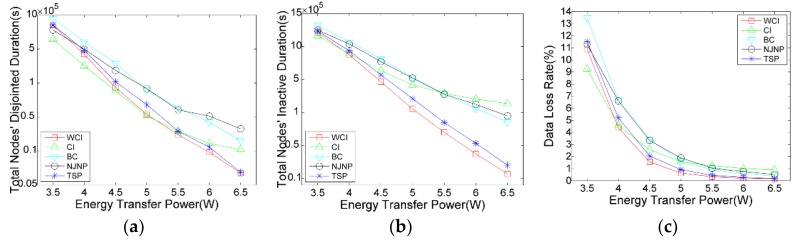
Impact of $p_{r}$ on the charging scheduling algorithms: (**a**) Total nodes’ disjointed time; (**b**) Total nodes’ inactive time; (**c**) Network’s data loss rate.

An important factor that affects the charger’s ability while performing charging tasks is the node’s received charging rate, which can determine the node’s full charging time. With the observation in Figure 8, when the charging rate is low, all of the five scheduling algorithms perform badly. This is because, with the lower charging rate, the charger takes more time to complete the charging tour, with the result that some high-energy-consumption nodes charged in the early time will run out of their energy before the charger’s next round visiting. When the charging rate increases, the performances of these algorithms, especially WCI and TSP, are improved. For example, when $p_{r}=5$, WCI’s total disjointed duration, inactive duration, and data loss rate is 72%, 70%, and 69% of that delivered by TSP, respectively. When compared with NJNP, the three ratios are reduced to 42%, 34%, and 34%, respectively. Note that CI outperforms in disjointed duration when the charging power is low, while it suffers inferior performance in total inactive duration.

## 6. Conclusions and Future Work

In this paper, we have proposed a novel mobile charger scheduling algorithm for WRSN. The proposed algorithm finds a charging tour for the mobile charger by maximizing the charging nodes’ total weighted criticality indexes which can measure the node’s connectivity contribution and energy criticality, subject to the traveling distance constraint of the mobile charger. Due to the NP-hardness of the problem, we then design a fast heuristic algorithm to solve it. Finally, we evaluate the performance of proposed algorithm against the NJNP and TSP scheduling algorithm through simulations. The proposed algorithm outperforms the others, particularly by reducing the network’s data loss. Besides this, we also compare the proposed algorithm with different rewards, which are the criticality index without energy criticality and betweenness centrality. From the result, we can verify the necessity and feasibility of the proposed weighted criticality index.

Some issues still exist that are worthy of further study. First, in the realistic environment, the battery life of a sensor node can be correlated to many issues, such as the node’s transmission range (near field or far field), application type (temperature reading, imaging, video surveillance, tracking, etc.), deployment environment (city, field, mountain, forest, etc.), routing structure (single-hop, multi-hop, etc.), sink type (mobile sink [32] or fixed sink) and so on. In this paper, we simplify the energy consumption model of the sensor nodes. It’s not very similar to the practical issues. Second, the charging efficiency of the WPT technology is related to the charging distance and antenna type. In this paper, we only consider the received charging rate of nodes, and neglect the power loss of the mobile charger. In order to improve the energy efficiency of the mobile charger, the charging distance between nodes and the mobile charger and the other antenna type of the WPT devices can also be considered. Finally, many obstacles in the practical environment exist which can cause trouble during the mobile charger’s movement. Designing a charging tour to avoid such obstacles is a new challenge in WRSN. In future work, we will focus on the charging model refinement and parameter consideration of the issues mentioned above to facilitate practical implementations.

## Figures and Tables

**Table 1:** List of notations.

Notation	Definition
N	node sets
$E_{max}$	the node’s battery capacity
$E_{min}$	the node’s minimum energy level for operation
$E_{i}$	node i’s residual energy level
v	the charger’s traveling speed
L	the charger’s traveling distance constraint
$p_{r}$	the node’s received charging rate
$T_{w}$	the period of time for the charger’s replenishment
$d_{s}$	the node’s sensing range
$d_{r}$	the node’s maximum transmission range
$e_{s}$	energy consumed to sense an event
$e_{t}$	energy consumed to transmit a packet
$e_{r}$	energy consumed to receive a packet
$e_{c}$	energy consumed to combine a packet

**Table 2:** Default parameters setting.

Parameters	Values
Network Size	100 × 100 m^2^
Number of nodes	100
$E_{max}$	1000 J
$E_{min}$	0 J
v	1 m/s
L	600 m
$p_{r}$	5 W
$T_{w}$	1000 s
$d_{s}$	10 m
$d_{r}$	25 m
$e_{s}$	0.15 mJ
$e_{t}$	5 mJ
$e_{r}$	1.6 mJ
$e_{c}$	0.05 mJ
Simulation time	100,000 s
